# Valorization of cricket, *Acheta domesticus* (Linnaeus, 1758), flour as a source of dietary protein in Japanese quail, *Coturnix japonica* (Temminck and Schlegel, 1849), farming

**DOI:** 10.5455/javar.2022.i598

**Published:** 2022-06-28

**Authors:** Francois Djitie Kouatcho, Razvan Mihail Radu Rusu, Bachirou Mohamadou, Bobga Aoudou, Ioan Mircea Pop, Marius Giorgi Usturoi, Léonard S. Ngamo Tinkeu

**Affiliations:** 1 Department of Science and Technology of Organic Agriculture, Faculty of Sciences / School of Chemical Engineering and Mineral Industries, University of Ngaoundéré, Ngaoundéré, Cameroon; 2 Department of Management of Animal Productions, Faculty of Food and Animal Sciences, Iaşi University of Life Sciences, Iaşi, Romania; 3 Department of Biological Sciences, Faculty of Sciences, University of Ngaoundéré, Ngaoundéré, Cameroon

**Keywords:** Carcass, cricket flour, fish meal, growth, substitution, Sudano-Guinean zone

## Abstract

**Objective:**

Quail production is ranked as an important alternative animal protein source in Cameroon. One of the main constraints of this production is the quality of feed, which lacks protein that is regularly supplied by fish meal. To avoid disagreements due to the constant shortage of fish meal, alternative protein sources are needed, and among them are crickets (*Acheta domesticus*). The goal of this study was to find out how well Japanese quails could be raised if fish meal was replaced with cricket meal.

**Materials and Methods:**

A total of 192, 4-week-old quails were divided into 12 similar sets of 8 females and 8 males. The Four experimental diets (T00, T15, T30, and T45) were all formulated based on the level (0%, 15%, 30%, and 45%, respectively) of fish meal substitution with cricket meal in the basal diet (crude protein: 20.18% and ME: 3,013.78 kcal/kg) and randomly assigned to three sets in a completely randomized design consisting of four treatments each repeated three times. Growth, carcass, and some reproduction parameters were collected. The data were analyzed using one-way analysis of variance and the Duncan test, with a significance level of 5%.

**Results:**

Cricket meal diets increased body weight in males (204.32 ± 5.69 gm for T45) and regardless of the sex (226.72 ± 29.45 gm for T30) compared to 184.17 ± 3.11 gm and 214.55 ± 32.77 gm for the control group, respectively. In females, substitution at 30% increased body weight (253.80 ± 6.48 gm), while 45% induced a reduction (216.67 ± 6.49 gm) when compared to the control group value (244.92 ± 6.07 gm). Carcass yield and the proportion of different parts were not significantly affected by the experimental diets. Liver proportions were significantly higher at 15% and 35% cricket meal incorporation compared to 45%. First songs and egg laying were recorded at 7 weeks with T15, which also led to improved egg laying performance compared to the other treatments. Ovaries were poorly developed in the T45 females compared to the other treatments.

**Conclusions:**

Cricket flour might be a good candidate as a locally available protein source to substitute fish meal in the Japanese quail’s diet at the finisher and reproductive stages, and the level of 30% seems to be more efficient.

## Introduction

The world production of animal feed passed the one billion ton mark in 2016. Because it can weigh up to 80% of the total cost of production, feed is the most expensive item in animal husbandry [1,2]. Fish meal is one of the main raw materials used to feed poultry and is also one of the main sources of protein [3]. Roughly 27% of sea fishery resources are used for processing into fish meal or fish oil without satisfying the needs of the livestock industry [3]. Overfishing is usually practiced to meet human and animal needs, and it exerts a high pressure on the aquatic ecosystem. The fact that Cameroon has to import all of its fish meal, the lack of stock, and the high price (more than 1 USD/kg) makes it hard for livestock farming in Cameroon and the Sudano-Guinean zone specifically to grow.

In this zone, there is a seasonal profusion of insects, such as crickets (*Acheta domesticus*), which are not part of the local diet and whose use as an additional source of protein in animal feed, in general, and in poultry farming, specifically, could not only help to reduce stock shortages but also enhance the value of local resources and even reduce production costs and competition between humans and animals for these resources. Given this observation and the fact that edible insects can be used in animal feed [4–6], the need for this study aimed at valorizing cricket meal in poultry farming, in general, and Japanese quail (Temminck and Schlegel, 1849), in particular, which has been of obvious interest among Cameroonian poultry farmers in recent years. Due to its low production cost associated with its small size, its resistance to the disease, rapid growth, and relatively short life cycle of up to 3–4 generations per year, and high egg production of 250–350 eggs/female/year [7–9], as well as the therapeutic virtues of these eggs [10], the expansion of this species has been accentuated. The general aim of this work is to contribute to the better profitability of quail husbandry by valorizing a locally available resource. Specifically, the effect of replacing fish meal with cricket meal on growth performance, carcass characteristics, and some reproduction parameters will be assessed in quails.

## Materials and Methods

### Ethical approval

Experimental protocols used in this study were endorsed by the ethics committee of the Faculty of Sciences of the University of Ngaoundéré, Cameroon, and complied strictly with internationally recognized standard ethical directives for the use and care of laboratory animals, as specified in the European Community Directive 86/609/EEC as of November 24, 1986.

### Study area

The study was carried out in Ngaoundéré, located in the Sudano-Guinean zone of Cameroon. The area is located between 6° and 8° degrees north latitude and between 10° and 16° degrees east longitude, at an average altitude of 1,000 m. The rainy season lasts 8 months (April–November), while the dry season lasts 4 months (December–March). The annual average rainfall is 1,500–1,800 mm, the annual average humidity is 64.1%–67.6%, and the temperatures range from 23°C to 32°C.

### Animal material, housing, and feeding

**Figure 1.**
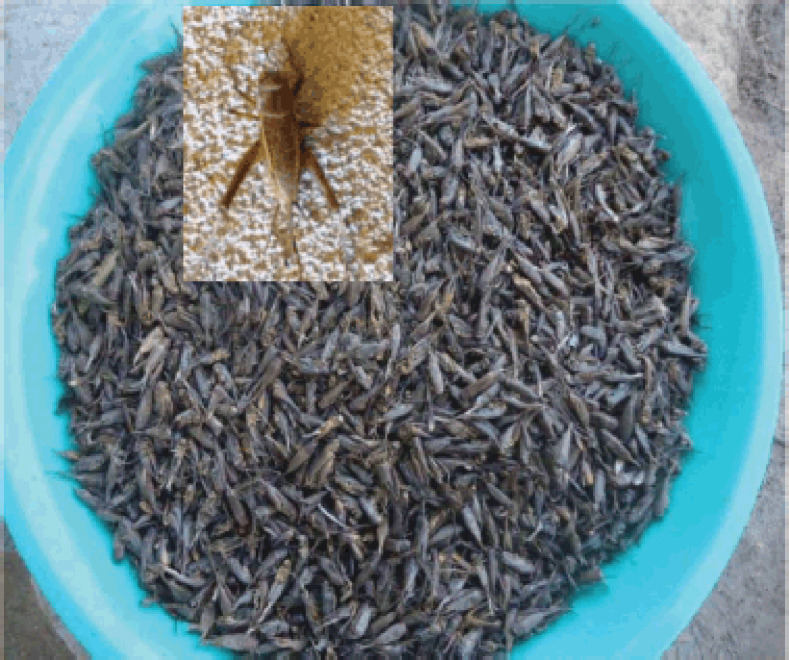
Crickets (*A. domesticus*) dried and ready to be ground.


The crickets (*A. domesticus*) used in the present trial were harvested in the locality of Ngaoundéré under street lamps at night. They were then dried (Fig. 1) the next day under the sun before being ground and stored in a dry, closed plastic container until they were used.

A total of 192 Japanese quail (96 females and 96 males) aged 28 days, with a mean weight of 62.79 ± 2.06 gm, was divided into 12 similar batches of 16 birds (8 females and 8 males). They were housed in cages made of board and wire mesh with a density of 28 birds per m^2^. A basic feed with 3,013.78 kcal/kg metabolizable energy and 20.18% crude protein was used to make four experimental diets (T0, T1, T2, and T3) that varied in how much fish meal was replaced with cricket meal (0%, 15%, 30%, and 45%, respectively).

### Experimental design and trial management

Each of the four experimental diets was allocated randomly to three sets in a fully randomized design consisting of four treatments (substitution level) replicated three times each. Feed and water were supplied *ad libitum* throughout the study. Except for the feed, all birds were kept under similar husbandry conditions.

### Data collection and studied variables

During the 4-week trial, data were recorded weekly on feed intake (FI), body weight, weight gain, feed conversion ratio (FCR), laying rate, and external egg characteristics. For each week, feed was weighed and distributed on a daily basis. The leftovers from each experimental unit were weighed every 7 days using an electronic scale, with a 5,000 gm capacity and 1 gm accuracy. The weekly FI was the difference between the amount of feed distributed during the week and the leftovers of the same week. Initially, and every 7 days thereafter, fasted birds were weighed in the morning using an electronic scale, with a 2,000 gm capacity and 0.1 gm accuracy. Weekly weight gain was derived as the difference between two consecutive body weights. The weekly FI and weight gain data for the same period were used to calculate the FCR as follows:

$$\mathrm{FCR}\;=\;\frac{\mathrm{Feed}\;\mathrm{int}\mathrm{ake}\;(\mathrm{gm})}{\mathrm{Average}\;\mathrm{weight}\;\mathrm{gain}\;(\mathrm{gm})}$$

### Carcass characteristics

At the end of the growth phase (7 weeks), nine males and nine females per treatment (three per batch) were fasted, sacrificed, and then characterized in accordance with the process described by Genchev and Mihaylov [11] for the evaluation of carcass characteristics. Data collected on the carcass, liver, heart, gizzard, head, thigh, back, wings, and leg weight were calculated using the following: 

$$\mathrm{Carcass}\;\mathrm{yield}\mathrm{(\%)}\;=\;\frac{\mathrm{Carcass}\;\mathrm{weoght}\;(\mathrm{gm})}{\mathrm{Body}\;\mathrm{weight}\;(\mathrm{gm})}\times \;100$$

Relativeweightofpartsororgans(%)=weightofpartsororgansBodyweight(gm)×100

### Reproductive characteristics

**Testes characteristics**

After quails were sacrificed, the testes were collected and weighed together and individually using an electronic scale, with 500 gm capacity and 0.01 gm accuracy. Then, the diameter and height of each testicle were measured using a digital caliper, with a span of 150 mm and 0.01 mm accuracy. The collected data were evaluated as follows:

Testicular shape index = Diameter (mm) / Height (mm)


$$\mathrm{Testis}\;\mathrm{weight}\;\mathrm{ratio}\;=\;\frac{\mathrm{Left}\;\mathrm{testis}\;\mathrm{weight}\;(\mathrm{gm})}{\mathrm{Right}\;\mathrm{testis}\;\mathrm{weight}\;(\mathrm{gm})}$$

$$\mathrm{Gonado}\;-\;\mathrm{somatic}\;\mathrm{index}\mathrm{(\%)}\;=\;\frac{\mathrm{Testis}\;\mathrm{weight}\;(\mathrm{gm})}{\mathrm{Live}\;\mathrm{weight}\;(\mathrm{gm})}\times \;100$$

**Egg laying rate and characteristics of the eggs**

During 2 weeks and using the equipment used to characterize testes, eggs obtained in each batch were collected daily and enumerated to calculate the weekly laying rate as follows:


$$\mathrm{Weekly}\;\mathrm{laying}\;\mathrm{rate}\;\mathrm{(\%)}\;=\;\frac{\mathrm{Number}\;\mathrm{of}\;\mathrm{eggs}\;\mathrm{laid}}{\mathrm{Number}\;\mathrm{of}\;\mathrm{females}\;\times \;7}\times \;100$$

### Statistical analysis

The data were presented as the mean ± standard deviation of the mean. A one-way analysis of variance was used, based on the general linear model, to compare the means of the different variables. In the case of differences between the treatments, means were separated with the Duncan test at the 5% significance level. For these analyses, IBM SPSS Statistics 25.0 software was used.

## Results

### Average production performances based on the level of substitution of fish meal with cricket meal

The effect of fish meal substitution with cricket meal on the average growth performance of quails at 8 weeks (Table 1) shows that, regardless of the sex, FI was significantly lower in the 15% and 45% cricket meal substituted batches than in the control and 30% of the cricket meal substituted batches, which were otherwise similar.

Regardless of the sex, the significantly higher body weights and weight gain were found with 30% substitution (T2) compared to the values of the other treatments, which were otherwise similar. In females, body weights and weight gain of the control and T2 treatments (30% substitution) were comparable and considerably higher (*p* < 0.05) than those of the 15% and 45% substitution treatments, which were also comparable. The meal was associated with a significant increase in body weight and average weight gain (AWG) in males compared to the values recorded with the control. Body weight and weight gain at the 45% substitution level were significantly higher than T1 (15%) but similar to 30%, which in turn was similar to T1.

The lowest FCR was recorded at T2 in females, regardless of the sex. When compared to the control, when fish meal was used instead of cricket meal, the FCR decreased overall.

### Feed intake

**Figure 2.**
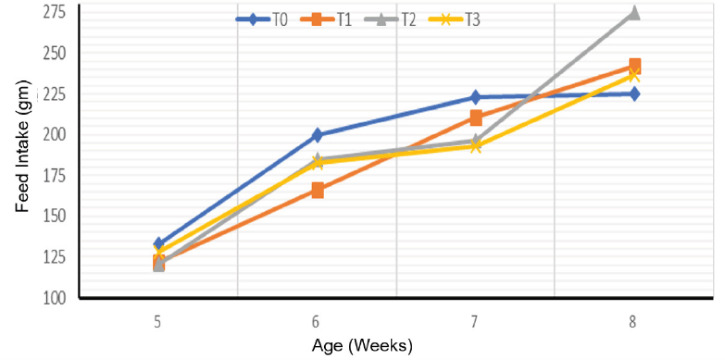
Weekly evolution of FI according to the experimental diets.

FI increased throughout the grow-out phase, regardless of the treatment (Fig. 2). Substitution of fish meal with cricket meal induced a significant reduction in FI between the fifth and seventh week of age compared to the control. At week 5, the FI of the T3 treatment (45%) was higher than that of the treatments at the substitution levels of 15% (T1) and 30% (T2). At week 6, T2 and T3 FI were similar and significantly (*p* < 0.05) higher than T1. In the seventh week, ingestion of 15% cricket meal in the feed (T1) induced a higher FI compared to that of the 30% and 45% inclusion of cricket meal in the feed. At the end of the trial, the highest FI was obtained with treatment T2 compared to the other treatments. Control treatment was significantly (*p* < 0.05) lower than T1 but similar (*p* > 0.05) to the T3 treatment.

**Table 1.** Average production performances of quails at 8 weeks of age as a function of the substitution level of fish meal with cricket meal.

Characteristic	Average production performances				
	Experimental diets	FI (gm)	Body weight (gm)	AWG (gm)	FCR
Male	T0 (00%)		184.17 ± 3.11^a^	30.34 ± 0.77^a^	6.43 ± 0.42^b^
	T1 (15%)		194.15 ± 3.91^b^	32.84 ± 0.97^b^	5.64 ± 0.20^a^
	T2 (30%)		199.65 ± 5.28^b^^c^	34.21 ± 1.32^b^^c^	5.68 ± 0.36^a^
	T3 (45%)		204.32 ± 5.69^c^	35.38 ± 1.42^c^	5.23 ± 0.17^a^
	Average		195.57 ± 8.78	33.19 ± 2.19	5.74 ± 0.52
Female	T0 (00%)		244.92 ± 6.07^b^	45.53 ± 1.51^b^	4.28 ± 0.17^ab^
	T1 (15%)		224.67 ± 5.94^a^	40.47 ± 1.48^a^	4.57 ± 0.16^b^^c^
	T2 (30%)		253.80 ± 6.48^b^	47.75 ± 1.62^b^	4.07 ± 0.28^a^
	T3 (45%)		216.67 ± 6.49^a^	38.47 ± 1.62^a^	4.81 ± 0.21^c^
	Average		235.01 ± 16.42	43.05 ± 4.10	4.43 ± 0.34
Combined	T0 (00%)	780.71 ± 29.50^b^	214.55 ± 32.77^a^	37.94 ± 8.19^a^	5.36 ± 1.18^b^
	T1 (15%)	740.54 ± 6.00^a^	209.41 ± 16.96^a^	36.65 ± 4.24^a^	5.11 ± 0.59^ab^
	T2 (30%)	776.37 ± 31.22^b^	226.72 ± 29.45^b^	40.98 ± 7.36^b^	4.87 ± 0.91^a^
	T3 (45%)	740.40 ± 9.92^a^	210.50 ± 8.69^a^	36.92 ± 2.17^a^	5.02 ± 0.28^ab^
	Average	759.51 ± 28.69	215.29 ± 23.86	38.12 ± 5.96	5.09 ± 0.79

a, b: in the same column and for the same sex, values with the same superscript are similar (*p*> 0.05).

### Body weights

**Figure 3.**
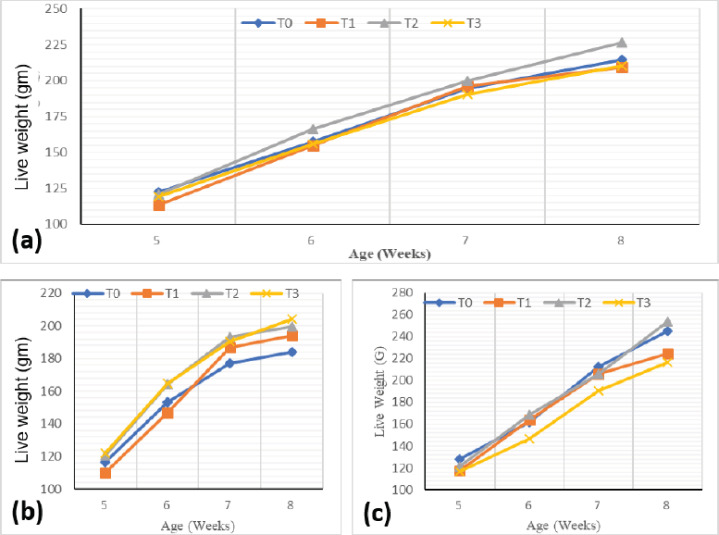
Weekly evolution of the body weight of quails as a function of experimental diets. (a) Combined live weight. (b) Male. (c) Female.

The weekly body weight assessment of quails showed the same pattern, regardless of the substitution rate of fish meal with cricket meal and sex (Fig. 3). It showed an increase in body weight with age, regardless of the sex, and higher values throughout the trial (Fig. 3a) induced by 30% (T2) substitution of fish meal with cricket meal in quail feed.

In males (Fig. 3b), however, at the beginning of the trial, FI at 30% (T2) and 45% (T3) substitution induced significantly (*p* < 0.05) higher body weight values compared to the 15% substitution treatment (T1). T2 and T3 treatments were significantly higher at week 6. At week 7, the control treatment induced lower but similar values. At the end of the trial period, the significantly highest body weight value was recorded with treatment T3 (204.32 ± 5.69 gm) compared to the control and T1. The significantly lower value was obtained with the control treatment. In females (Fig. 3c), it was found that at week 5, the mean body weight of treatment T0 (127.97 ± 4.23 gm) was significantly (*p* < 0.05) higher than treatments T1 and T3, but all were similar to treatment T2. For weeks 6 and 7, the weight of treatment T3 was significantly lower than the other treatments, which were otherwise comparable (*p* > 0.05) to each other. In the last week, the body weights of control and T2 treatments were significantly (*p* < 0.05) higher than those of T1 and T3 treatments. Ingestion of a feed in which 45% of the fish meal was substituted by cricket meal (T3) induced the lowest body weight throughout the trial, although it was comparable to T1.

### Average weight gain

**Figure 4.**
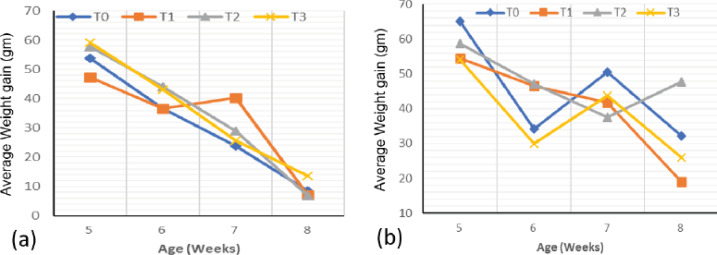
Evolution of the AWG (gm) of quails according to the experimental diets. (a) Male. (b) Female.

AWG in quails decreased with age (Fig. 4), although in females (Fig. 4b) it was irregular. In males (Fig. 4a), at the beginning of the trial, a significantly (*p* < 0.05) lower weight gain was recorded with treatment T1 (47.16 ± 4.60 gm) compared to T2 and T3, although they remained similar (*p* > 0.05) to the control treatment. At week 8, the T3 treatment exhibited a significantly (*p* < 0.05) higher mean weight gain value than the other treatments. At the fifth week, in females, the control batch had a significantly (*p* < 0.05) higher weight gain than treatments T1 and T3. At the end of the trial, treatment T2 had a significantly (*p* < 0.05) higher weight gain (47.75 ± 2.40 gm) than the control (32.22 ± 3.14 gm), which in turn was higher than T3. A significantly (*p* < 0.05) lower value of the AWG was recorded in the T1 treatment (19.02 ± 1.01 gm).

### Feed conversion ratio

**Figure 5.**
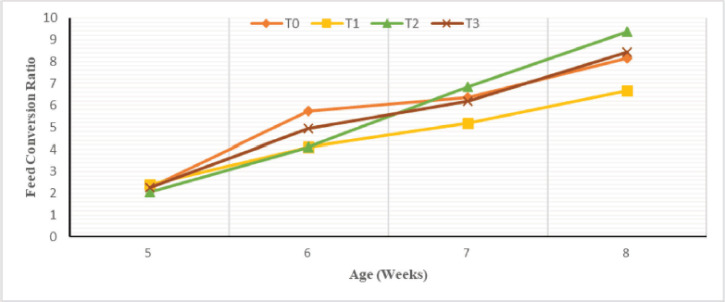
Weekly evolution of the FCR according to the experimental diets.

The evolution of the FCR, regardless of the sex, according to the level of substitution of fish meal with cricket meal is shown in Figure 5. It can be seen that the FCR increases with age. At week 5, the 15% substitution treatment was significantly (*p* < 0.05) higher than the T2 FCR. At week 6, the control treatment was significantly higher than the T1 and T2 treatments but remained similar to T3. The latter were, furthermore, similar to the values of the T3 treatment. A reverse trend was observed at 7 and 8 weeks where the FCR of treatment T2 was significantly greater than that of T1.

### Carcass characteristics and proportions of parts and organs as a function of the level of substitution of fish meal with cricket meal

**Carcass characteristics**

Carcass characteristics and proportions of parts relative to body weight as a function of the level of substitution of fish meal with cricket meal are summarized in Table 2. Except for neck proportions, carcass yield and part proportions were not significantly affected by the level of substitution of fish meal with cricket meal. Carcass yield ranged from 69.52% (control) to 72.00% (T2) in males and from 62.15% (control) to 69.30% (T3) in females. Regardless of the sex, the lowest carcass yield was recorded with the control treatment (65.83% ± 4.10%) and the highest with the T3 treatment (69.93% ± 1.68%). However, there was an increase in carcass yield in birds fed cricket meal. The same trend was generally observed with the relative weights of the wings, back, and thighs. Even though there were no big differences in the values of the back, the proportions of this part got smaller when the cricket meal was used.

**Relative weight of some organs in relation to body weight of quail**

The effect of the substitution level of fish meal with cricket meal on the relative weight of some organs of quail is presented in Table 3. It can be seen that the proportions of abdominal fat and heart were not significantly affected by the substitution level. The proportions of liver in cricket meal treatments were found to be similar to those in the control and an ingestion of the feed with 45% (T3) substitution of fish meal with cricket meal induced significantly lower liver proportions than in 15% and 30% substitution level treatments. The significantly higher proportions of gizzard were found in the T3 treatment compared to the other treatments, which were otherwise similar.

Effect of fish meal substitution with cricket meal on some reproductive parameters in quail.

**Testes characteristics**

The effect of substituting fish meal with cricket meal on testicular characteristics is summarized in Table 4. Although broadly similar, it can be seen that the relatively highest testes weights were recorded with the control compared to the other treatments. This was also the case for the gonadosomatic index, where the highest value (1.38% ± 0.16%) was recorded with the control group and the lowest with those substituted at 15% and 45% with cricket meal. Testicular height and diameter were also not affected by the substitution rate. Even though the shape index was higher for the T3 treatment (45%) than for the other treatments, this parameter was the same at all substitution levels.

**Table 2.** Carcass characteristics of quails as a function of the substitution rate of fish meal with cricket meal.

Characteristics (%)	Gender	Experimental diets (level of substitution)			
		T0 (00%)	T1 (15%)	T2 (30%)	T3 (45%)
Carcass yields	Male	69.52 ± 1.10^a^	70.71 ± 3.33^a^	72.00 ± 1.21^a^	70.57 ± 1.85^a^
	Female	62.15 ± 0.26^a^	65.47 ± 3.23^a^	63.15 ± 6.62^a^	69.30 ± 1.57^a^
	Combined	65.83 ± 4.10^a^	68.09 ± 4.10^a^	67.58 ± 6.45^a^	69.93 ± 1.68^a^
Chest	Male	25.64 ± 0.95^a^	25.97 ± 2.41^a^	26.89 ± 1.70^a^	25.80 ± 1.39^a^
	Female	22.59 ± 2.04^a^	23.17 ± 1.73^a^	23.07 ± 3.81^a^	26.22 ± 3.77^a^
	Combined	24.11 ± 2.19^a^	24.57 ± 2.43^a^	24.98 ± 3.37^a^	25.80 ± 1.39^a^
Thigh	Male	15.69 ± 0.13^a^	16.28 ± 0.75^a^	17.38 ± 1.21^a^	16.61 ± 1.04^a^
	Female	14.82 ± 0.35^a^	16.06 ± 1.29^a^	14.70 ± 1.51^a^	16.39 ± 1.95^a^
	Combined	15.26 ± 0.53^a^	16.17 ± 0.96^a^	16.04 ± 1.91^a^	16.50 ± 1.40^a^
Wings	Male	5.38 ± 0.62^a^	5.99 ± 0.31^a^	5.92 ± 0.48^a^	5.37 ± 0.49^a^
	Female	4.89 ± 0.36^a^	5.34 ± 0.57^a^	5.13 ± 0.40^a^	6.06 ± 0.42^a^
	Combined	5.13 ± 0.52^a^	5.67 ± 0.54^a^	5.52 ± 0.59^a^	5.72 ± 0.56^a^
Head	Male	3.06 ± 0.19^a^	3.46 ± 0.19^a^	3.34 ± 0.18^a^	3.05 ± 0.17^a^
	Female	2.51 ± 0.21^a^	2.62 ± 0.13^a^	2.63 ± 0.14^a^	3.10 ± 0.20^a^
	Combined	2.79 ± 0.35^a^	3.04 ± 0.48^a^	2.98 ± 0.41^a^	3.08 ± 0.17^a^
Leg	Male	1.46 ± 0.06^a^	1.59 ± 0.22^a^	1.53 ± 0.12^a^	1.55 ± 0.19^a^
	Female	1.46 ± 0.12^a^	1.46 ± 0.11^a^	1.37 ± 0.24^a^	1.54 ± 0.29^a^
	Combined	1.46 ± 0.08^a^	1.53 ± 0.17^a^	1.45 ± 0.19^a^	1.54 ± 0.219^a^
Neck	Male	6.75 ± 0.68^ab^	7.39 ± 1.08^b^	6.91 ± 1.53^ab^	5.97 ± 0.99^a^
	Female	6.35 ± 0.49^ab^	7.66 ± 0.73^b^	6.69 ± 0.98^ab^	6.37 ± 0.89^a^
	Combined	6.55 ± 0.58^ab^	7.52 ± 0.84^b^	6.80 ± 1.16^ab^	6.17 ± 0.87^a^
Back	Male	16.06 ± 1.05^a^	14.37 ± 1.59^a^	13.96 ± 0.93^a^	13.86 ± 0.50^a^
	Female	13.49 ± 2.03^a^	12.35 ± 0.19^a^	12.72 ± 2.26^a^	13.86 ± 0.50^a^
	Combined	14.77 ± 2.02^a^	13.36 ± 1.50^a^	13.34 ± 1.69^a^	13.86 ± 0.50^a^

a, b: in the same line, values with the same superscript are not significantly different (*p *> 0.05).

**Table 3.** Relative weight of some organs in relation to the body weight of quails.

Characteristics	Gender	Experimental diets (level of substitution)			
		T0 (00%)	T1 (15%)	T2 (30%)	T3 (45%)
Liver	Male	1.45 ± 0.39^ab^	2.10 ± 0.44^b^	2.06 ± 0.46^b^	1.71 ± 0.12^a^
	Female	3.05 ± 0.63^ab^	3.06 ± 0.45^b^	2.75 ± 0.23^b^	1.93 ± 0.12^a^
	Combined	2.24 ± 0.99^ab^	2.58 ± 0.60^b^	2.40 ± 0.50^b^	1.82 ± 0.16^a^
Heart	Male	0.91 ± 0.04^a^	0.86 ± 0.08^a^	0.83 ± 0.09^a^	0.80 ± 0.16^a^
	Female	0.77 ± 0.11^a^	0.79 ± 0.11^a^	0.71 ± 0.15^a^	0.77 ± 0.04^a^
	Combined	0.84 ± 0.11^a^	0.83 ± 0.09^a^	0.77 ± 0.13^a^	0.78 ± 0.11^a^
Guizard	Male	1.76 ± 0.37^a^	1.95 ± 0.30^a^	1.77 ± 0.18^a^	2.22 ± 0.38^b^
	Female	1.63 ± 0.20^a^	2.00 ± 0.32^a^	1.32 ± 0.04^a^	2.68 ± 0.70^b^
	Combined	1.70 ± 2.27^a^	1.97 ± 0.28^a^	1.55 ± 0.27^a^	2.45 ± 0.56^b^
Abdominal fat	Male	1.00 ± 0.53^a^	0.44 ± 0.40^a^	0.89 ± 0.65^a^	1.13 ± 0.47^a^
	Female	2.16 ± 0.32^a^	1.84 ± 0.50^a^	1.37 ± 0.10^a^	1.15 ± 0.52^a^
	Combined	1.58 ± 0.74^a^	1.14 ± 0.87^a^	1.13 ± 0.49^a^	1.14 ± 0.45^a^

a, b: in the same line, values with the same superscript are not significantly different (*p *> 0.05).

**Table 4.** Testicular characteristics as affected by the substitution rate of fish meal with cricket meal.

Characteristics		Experimental diets (substitution rate)			
		T0 (00%)	T1 (15%)	T2 (30%)	T3 (45%)
Weight	Left	1.32 ± 0.01^a^	0.72 ± 0.49^a^	1.15 ± 0.40^a^	0.89 ± 0.73^a^
	Right	1.36 ± 0.17^a^	0.72 ± 0.50^a^	1.02 ± 0.32^a^	0.98 ± 0.81^a^
	Total	2.69 ± 0.16^a^	1.44 ± 0.99^a^	2.17 ± 0.71^a^	1.88 ± 1.54^a^
Testicular left/right ratio		0.98 ± 0.12^a^	1.01 ± 0.13^a^	1.12 ± 0.12^a^	0.96 ± 0.10^a^
Gonadosomatic index		1.38 ± 0.16^a^	0.81 ± 0.62^a^	1.08 ± 0.46^a^	0.83 ± 0.69^a^
Height	Left	22.47 ± 0.19^a^	17.86 ± 4.64^a^	22.22 ± 1.96^a^	19.30 ± 9.83^a^
	Right	22.40 ± 2.07^a^	17.47 ± 3.41^a^	20.39 ± 2.90^a^	18.30 ± 9.77^a^
	Average	22.44 ± 1.13^a^	17.67 ± 4.02^a^	21.31 ± 2.43^a^	18.8 ± 9.8^a^
Diameter	Left	12.49 ± 1.62^a^	9.52 ± 1.76^a^	12.12 ± 1.63^a^	11.66 ± 6.18^a^
	Right	12.64 ± 1.35^a^	10.03 ± 1.28^a^	12.31 ± 1.13^a^	12.92 ± 6.81^a^
	Average	12.57 ± 1.49^a^	9.78 ± 1.52^a^	12.22 ± 1.38^a^	12.29 ± 6.50^a^
Shape index	Left	0.55 ± 0.07^a^	0.54 ± 0.05^a^	0.55 ± 0.05^a^	0.59 ± 0.04^a^
	Right	0.57 ± 0.11^a^	0.58 ç± 0.10^a^	0.61 ± 0.13^a^	0.70 ± 0.06^a^
	Average	0.56 ± 0.09^a^	0.56 ± 0.07^a^	0.58 ± 0.09^a^	0.65 ± 0.05^a^

a, b: in the same line, values with the same superscript are not significantly different (*p *> 0.05).

**Laying performances**

**Figure 6.**
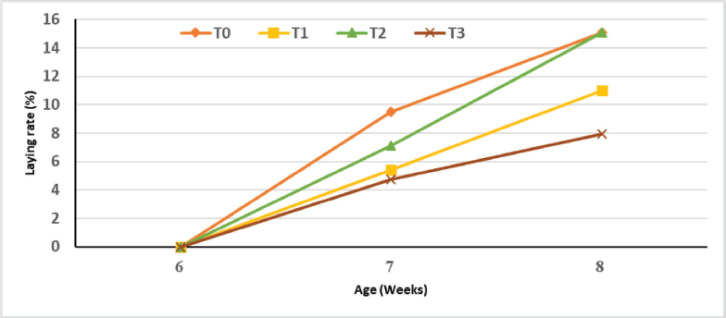
Evolution of the laying rate of quails according to the experimental diets.

The evolution of laying performance, regardless of the sex, according to the level of substitution of fish meal with cricket meal is shown in Figure 6. It can be seen from the figure that a higher laying rate at the beginning of the laying period of quails was recorded with the control group. The 30% (T2) substitution of fish meal with cricket meal in the quail feed presented the same laying rate as the control in 8-week-old females. It has also been noticed that a 45% substitution rate induced the worst laying rate during the trial period.

## Discussion

With the exception of treatment T2, where FI was the same as the control, there was a significant reduction in the treatments substituted at 15% and 45% compared to the control treatment. These results corroborate those of Deshpande et al. [13], who reported a decrease in FI and body weight gain in broiler diets involving a 50%–100% substitution of fish meal with silkworm meal. However, our observations are opposite to those of Liu and Lian [14], who replaced 20% and 40% of fish meal with grasshopper meal in broiler diets without any effect on FI and weight gain. Studies have also been carried out on Japanese quails during the egg-laying phase by Khan [15], who recorded an increase in FI by substituting the basic feed from 5% to 15%. These variations could be due to the genetic material, growth phase, or even the quality of the sources and processes used to obtain the insect powder. Indeed, the characteristics of the insect meal vary with the type of insect, as well as the drying and grinding processes that induce acceptability and selective growth among birds [16–18].

Except for the males, in which body weight increased significantly with the level of substitution of fish meal with cricket meal, treatment T2 (30%) induced in females and regardless of the sex, body weights were significantly higher than those of the other treatments and would reflect better feed assimilation due to a level of protein that would be optimal in this diet. The results also show that males seem to value cricket meals better than females. The values obtained in this study are consistent with the results of studies conducted by Djitie et al. [19] and De Lemos et al. [20] on the impact of the level of crude protein on quail growth in the finisher period in the highlands of Cameroon and the effect of some feed formulas on the zoological technical performance and biochemical profile of Japanese quails. This would indicate that this local protein source is suitable for feeding growing quails. Females fed 45% cricket meal had a lower body weight throughout the trial. Several studies have shown that increasing the protein content of the diet above a certain level in growing animals would usually have a negative effect on the weight of the animals, on both protein synthesis [21] and hormonal balance [22], due to the high fiber content.

In males, regardless of the sex, substitution of fish meal with cricket meal induced a significant decrease in the FCR as compared to the control group. A similar trend was recorded by Fitroh et al. [18], who noted a reduction in FCR by substituting cricket meal (*A. domesticus*) with the basal diet of 5%–15% in laying quails. A similar trend was recorded by Fitroh et al. [18], who noted a reduction in FCR by substituting cricket meal (*A. domesticus*) with 5%–15% of the basal diet in laying quails. Similarly, the work of Permatahati et al. [23], on the effect of cricket (*Gryllus bimaculatus*) meal on the production and physical quality of Japanese quail eggs, is noteworthy. These observations would indicate a better feed conversion in the groups considered. Indeed, the lower the FCR, the better the feed assimilated [24].

A much higher FCR was recorded for the control batches for the whole growth period, from 3.7 to 10.2 [25–27], but also lower FCR (from 2.72 to 3.32) by Bonos et al. [28], and a similar FCR to our control batch [20,29]. According to Kanyinji and Moonga [30], the increase in FCR may be a consequence of wastage and its reduction may be related to high fiber content. According to Reda et al. [31], factors influencing the FCR are environmental temperature, genetics, body weight, feed nutrient content, and gender differences due to variation in FI.

Although broadly similar, carcass yields increased relatively with the level of substitution. Similar trends were recorded by Cullere et al. [32]and Schiavone et al. [17], who, working on the effect of using black soldier fly larvae as a dietary protein source on the carcass and meat characteristics of broiler quail, and the use of defatted black soldier fly meal as a source of feed protein on broiler carcass characteristics and breast quality, respectively, noted a substantial increase in carcass yield when using black soldier fly larvae as a partial source of feed protein. These observations could reflect a higher protein content of insects compared to conventional protein sources, for instance, fish meal and soybean meal. Indeed, according to Józefiak and Engberg [33], the insect group comprises the largest variety of species in the world, and suitable species affording high concentrations of sulfur and protein-containing amino acids have been identified and can be successfully exploited in poultry feed.

Carcass yield values in our study were higher than the ones recorded with the control and in various quail species, including Pharaoh quails at 35 and 42 days by Wilkanowska et al. [34], meadow quails at 49 days by Vali [35],and Japanese quails between 35 and 56 days [35–37]. Furthermore, our values are close to those reported by Djitie et al. [19] for quails in the West Cameroon highlands and Seyed-Alireza et al. [38] for Japanese quails. The carcass yields of the T0 and T1 treatments of males were also similar to those noted by Baylan et al. [39] and Bonos et al. [28] at 35 and 42 days, respectively. These observations suggest that carcass yields are primarily affected by sex [19,37] and age of the animals rather than genetic type, as Vali [35] reported carcass yields of 60.13% and 60.37% at 49 days for Japanese quails and grassland quails, respectively. Fish meal seems to have at least the same effect on the number of quail carcasses that cricket meal does.

Although similar, breast, thigh, wing, and head proportions were relatively higher in animals fed cricket meal. This could reflect the higher nutrient content of the cricket meal compared to fish meal.

Although not significant, the proportions of the different parts of the carcass were relatively higher in quails consuming a feed containing cricket meal. This trend is contrary to that of Marareni and Mnisi [40], who found a reduction in the values of these components in their work on the study of growth performance, serum biochemistry, and meat quality in Jumbo quails given a diet comprising mopane worm meal (*Imbrasia belina*). However, this is not the case for the work of Cullere et al. [32], Hatab et al. [41], and Woods et al. [42], who fed birds with black soldier fly or *Hermetia illucen* larvae collected from different substrates and noted an increase in the values of the carcass proportions.

The proportions of the wishbone and back were generally lower than those noted by the majority of authors, regardless of the species and age of the quails, but the proportions of the head were higher than those obtained by Djitie et al. [19] at 49 days in the same species. These observations suggest that with a specific genetic type, the values of these parameters would be comparable, regardless of age, as confirmed by the work of Wilkanowska and Kokoszynski [43], who reported comparable values for the proportions of the wishbone and the back in Pharaoh quails between 33 and 42 days. From our results, it appears that cricket meal used up to 30% independently of sex relatively increased liver proportions, which subsequently dropped to 45% use. A reduction in relative heart weight and abdominal fat was also observed. These results are contradictory to those of Schiavone et al. [17], who supplemented broilers with defatted black soldier fly meal and noted a reduction in liver proportions. At 15% supplementation, they noted an increase in liver proportions.

Liver proportions were significantly affected by cricket meal at 15% and 30%. This is in line with the work of De Lemos et al. [20] at 30% FT on the effect of some dietary formulations on quails. According to Akiba and Matsumoto[44], high fiber diets decrease hepatic lipogenesis, leading to a reduction in hepatic lipid deposition and fatty acid synthesis from glucose. Contrary to our work, Djitie et al. [19], in their work on protein requirements of growing and breeding quails, obtained this result with 22% protein content in their diet. For them, these observations could reflect the hyperactivity of these organs compared to those of the other treatments. If we consider that the protein level contained in the T1 and T2 treatments is higher than the quail’s needs in finishing, the liver, which is an organ of detoxification of the organism, would act and its hyperactivity would lead to its hypertrophy [19].

The testicular weight noted with the control treatment is higher than those observed by Vatsalya and Kashmiri[45], but those obtained with the T2 treatment (30%) at 56 days are higher than the latter and those obtained by Wilkanowska et al. [34] on Pharaoh quails. This can be justified by the fact that the latter’s readings were taken on animals younger than ours, as testicular weights and volumes generally increase with age until puberty and reach maximum values at the sexual period [9,46]. The present study showed that the left testis weight is almost always more developed than that of the right testis, which is consistent with previous studies in quails [19]and birds [9,45,47]. The rapid testicular growth in the quails in our trial is thought to be due to the high propagation of Sertoli cells. Their increase in size results in the expansion of the seminiferous tubules, which translates into a progressive rise in testicular mass and volume [9,48].

In our trial, the first eggs and songs were observed with the T1 (15%) females aged between 6 and 7 weeks, in contrast to the observations made by Hassan et al. [49] at 50 days with his control batch. Sexual maturity was observed between 60 and 62 days by Retes et al. [25]. The sexual maturity of Japanese quails and other animals can be influenced by genetic factors, body weight, and other factors [50–53]. For De Lemos et al. [20], anti-nutritional factors are the causes of a delay in growth and are responsible for a decrease in weight and, consequently, an effect on the delay of sexual maturity.

The substitution of fish meal with cricket meal had effects on egg weight. It was noted that from the eigth week of our study, the highest egg weight was recorded with a 30% substitution rate, which otherwise would have had the highest feed consumption values, in accordance with the work of De Lemos et al. [20]. The egg production of treatment T2 (30% substitution rate) is close to that of the control treatment and equal to the latter at 8 weeks, the low rate of laying observed in our study, according to Retes et al. [25], who attributed the low egg production to the fact that the calculation started from the first egg laid. According to De Lemos et al. [20], the low laying rate could be due to the fact that the quails used are not genetically selected for laying.

## Conclusion

This study revealed that fish meal can be substituted by cricket meal in quail farming for economic production and valorization of this unconsidered local resource. The substitution rate of 30% seems to be more appropriate to improve the growth and egg laying performance of quail as it induced higher body weight and lower FCR as compared to the control group. The 45% replacement of fish meal by cricket flour seems to not be suitable for early laying carriers in quails since the lowest laying rate was recorded with this rate. The study of the reasons for this poor performance at a relatively high level of substitution associated with the characterization of cricket meal is considered for further research. 

## List of Abbreviations

AWG, average weight gain; °C, degree Celsius; FCR, feed conversion ratio; FI, feed intake; g, gram; kcal, kilocalories; kg, kilogram; mm, millimeter; USD, United States dollar.
